# Limited Shared Variance among Measures of Cognitive Performance Used in Nutrition Research: The Need to Prioritize Construct Validity and Biological Mechanisms in Choice of Measures

**DOI:** 10.1093/cdn/nzab070

**Published:** 2021-04-26

**Authors:** Michael J Wenger, Diane M DellaValle, Lauren E Todd, Amy L Barnett, Jere D Haas

**Affiliations:** Department of Psychology, Cellular, and Behavioral Neurobiology, The University of Oklahoma, Norman, OK, USA; Division of Nutritional Sciences, Cornell University, Ithaca, NY, USA; Division of Nutritional Sciences, Cornell University, Ithaca, NY, USA; Nutrition Science, King's College, Wilkes-Barre, PA, USA; Division of Nutritional Sciences, Cornell University, Ithaca, NY, USA; Department of Psychology, Cellular, and Behavioral Neurobiology, The University of Oklahoma, Norman, OK, USA; Division of Nutritional Sciences, Cornell University, Ithaca, NY, USA

**Keywords:** memory, cognition, functional outcomes, measurement, attention

## Abstract

**Background:**

The literature on correlates of nutrition has seen an increase in studies focused on functional consequences at the levels of neural, perceptual, and cognitive functioning. A range of measurement methodologies have been used in these studies, and investigators and funding agencies have raised the questions of how and if these various methodologies are at all comparable.

**Objective:**

The aim was to determine the extent to which 3 different sets of cognitive measures provide comparable information across 2 subsamples that shared culture and language but differed in terms of socioeconomic status (SES) and academic preparation.

**Methods:**

A total of 216 participants were recruited at 2 US universities. Each participant completed 3 sets of cognitive measures: 1 custom-designed set based on well-understood laboratory measures of cognition [cognitive task battery (COGTASKS)] and 2 normed batteries [Cambridge Neuropsychological Test Automated Battery (CANTAB), Weschler Adult Intelligence Scale, fourth edition (WAIS-IV)] designed for assessing general cognitive function.

**Results:**

The 3 sets differed with respect to the extent to which SES and educational preparation affected the results, with COGTASKS showing no differences due to testing location and WAIS-IV showing substantial differences. There were, at best, weak correlations among tasks sharing the same name or claiming to measure the same construct.

**Conclusions:**

Comparability of measures of cognition cannot be assumed, even if measures have the same name or claim to assess the same construct. In selecting and evaluating different measures, construct validity and underlying biological mechanisms need to be at least as important as population norms and the ability to connect with existing literatures.

## Introduction

The literature on nutritional deficiencies and their amelioration has seen an increase in studies focused on consequences at the levels of neural, perceptual, and cognitive functioning, from both basic science and translational perspectives (1–6). Consider that in the 5 y between 2016 and 2020 (inclusive) >13,000 papers were published on some aspect of nutrition and cognition, at an average of >2600 papers per year (see **Table 1**; see the **Supplemental Material** for details on how these estimates were obtained). A range of measurement methodologies have been used in these studies, and investigators and funding agencies have raised the questions of how and if these various methodologies are at all comparable. Any sense of cumulative progress in this domain requires an understanding of the level of comparability across approaches. We present here, to our knowledge, the first and only controlled within-person comparison of different measurement approaches.

**FIGURE 1:**
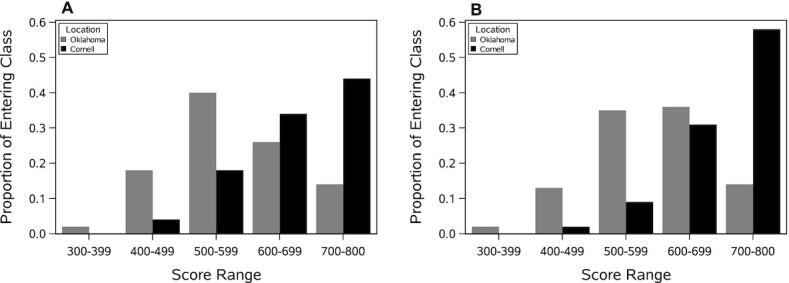
Distribution of SAT reading (A) and SAT mathematics (B) scores as a function of testing location. SAT, Scholastic Aptitude Test.

**TABLE 1:** Estimated number of papers published per year and cumulatively on nutrition and cognition from 2016 and 2020

Year	Total	Cumulative
2016	2210	2210
2017	2510	4720
2018	2350	7070
2019	2960	10,030
2020	3400	13,430

The Bill and Melinda Gates Foundation and Grand Challenges Canada commissioned a review of currently used measures in 4 domains of interest: cognitive abilities, social and behavioral development, motor skills, and home environment. The final report from this work (7) concluded that “there is no ‘one size fits all’” approach and that there is no identifiable “gold standard” for measuring functional outcomes in these domains. Although the report allows the range of measures to be grouped in terms of functional domains, appropriate populations, etc., it offers no guidance in terms of determining whether the various tools are in fact assessing comparable abilities and functions in comparable ways.

A review of the measures considered in that report reveals 3 general challenges to any attempt to assess comparability. First, the majority of the measures lack theoretical or biological specificity to the functions they propose to assess. For example, the measures of memory appropriate for adolescents or young adults considered in that report include general measures of intellectual performance as well as scales developed for application to career development. They span up to 6 of what the authors of the report identify as subdomains of general cognitive functioning, which relate only loosely to currently accepted scientific conceptions of memory (8) and have no apparent reference to brain systems and circuits that support memory (9). Second, a large number of the measures (including many that are timed) are administered manually, without appropriate instrumentation, and those that are administered with instrumentation either do not report or do not allow the precision and consistency of their measurements to be assessed. This is critical because differences across display and clock technologies can often lead to large variations in measured response latencies (10), especially when the differences of interest exist on the scale of milliseconds. Third, many applied studies (such as intervention studies) have a concern with the performance of specific populations with specific functional needs (e.g., factory workers, tea pluckers, students, etc.). The generality of the majority of the tests considered in that report a priori limits the relevance of what is measured to the functional needs of the population.

The lack of specificity with respect to the cognitive construct of interest and/or the biological underpinnings of that construct, the lack of concern with proper and precise instrumentation, and the lack of functional relevance to the population of interest all suggest that the results from the use of these kinds of measures may lead to muddled outcomes. And indeed, that is the case, as has been noted (11–13).

The present study was a controlled comparison of 2 general approaches in the form of 3 different test batteries. The first is one that we have used in a set of field studies of interventions designed to address the consequences of iron deficiency (1, 4, 14), referred to here as COGTASKS. The tasks used to assess cognitive performance in these studies were selected, in part, on the basis of the extent to which they rely on brain areas differentially sensitive to variation in iron (15, 16) and, in part, on the extent to which they assess functionally relevant abilities (14). The second approach is represented by 2 frequently used, normed batteries of cognitive functioning: the Cambridge Neuropsychological Test Automated Battery (CANTAB; Cambridge Cognition) (17–19), and the Weschler Adult Intelligence Scale, fourth edition (WAIS-IV; Pearson) (20–22). Critically, all 3 sets of measures included tasks that had the same name [e.g., go/no-go (GNG)] or that claim to measure the same cognitive construct (e.g., working memory). All 3 sets of measures were taken by 2 samples of healthy, nonclinical, college-aged women, one at The University of Oklahoma (OU) and the other at Cornell University (CU). The ability to acquire measurements at these 2 universities allowed us to quantify the patterns of shared and distinct variance using 2 samples that possess a common language and culture but that differ in 2 specific characteristics—socioeconomic status (SES) and general academic achievement—known to modulate a range of perceptual and cognitive measures (23). To our knowledge, although the questions of interest are important, the present study is the first and only one to perform a controlled, within-participants investigation of shared variance. Our predictions were that *1*) the shared variance across the tasks, even though many shared the same name or claim to investigate the same cognitive construct, would be low, and that *2*) the shared variance within task sets would be much higher than across task sets.

## Methods

### Subjects

A total of 216 women were recruited at 2 testing locations: half of the sample was recruited from the Norman, Oklahoma, campus of OU, and half of the sample was recruited from the Ithaca, New York, campus of CU. We restricted consideration to females on the basis of our primary interest in the effects of iron deficiency on cognition. All subjects had (self-reported) normal or corrected-to-normal vision and hearing, were proficient in written and spoken English, and reported unencumbered use of both hands. Subjects were compensated with a $50 gift card at the end of 3 d of participation.

### Study design

The study was designed as a 2 (location: OU, CU) × 3 (assessment: COGTASKS, CANTAB, WAIS-IV) factorial with assessment as a within-subjects variable. The ordering of test battery per subject was determined using a balanced Latin square, and the ordering of the tasks within each battery was fixed.

### Cognitive assessments

The 3 sets of cognitive assessments were administered on 3 consecutive days. All tasks were administered by research assistants trained to a common standard by MJW and DMDV, who also performed random periodic checks for consistency of procedure.

#### COGTASKS

All of the tasks were developed and programmed by MJW using public-domain software (10) that allowed for highly accurate timing of stimulus displays and behavioral responses (±1 ms); all programs and stimuli are freely available on request. Each of the tasks have long histories in the experimental study of cognition, with some dating back to the 19th century (24). This is to say that, although the tasks do not have associated norms in the traditional sense, there is a long literature than can be consulted for normative patterns. Brief descriptions of the tasks are provided here, with procedural details presented in the Supplemental Material.

The simple reaction time (SRT) task provides an estimate of the speed of the simplest possible behavioral response to a visual stimulus. The GNG task provides an estimate of the efficiency of sustained attention and the speed of attentional capture in the absence of a need to filter competing information. The attentional network task (ANT) (25) provides an estimate of the effectiveness of 3 components of attention: alerting (low-level attentional capture), orienting (midlevel spatial selective attention), and conflict (high-level selection). The Sternberg memory search (SMS) task (26) estimates the speed and accuracy with which immediate visual memory can be searched. The composite face effect (CFE) task (27) estimates the extent to which information from immediate perception and memory can be effectively coordinated. The cued recognition task (CRT) follows a modified (28) version of a classic (24) visual recognition memory task that estimates the speed, accuracy, and efficiency of recognition based on short-duration visual memory.

#### CANTAB

The CANTAB measures specific aspects of cognition, including memory and learning. The SRT task provides an estimate of the speed of the simplest possible behavioral response to a visual stimulus. The affective go/no-go task (AGNG) evaluates latency, error, and bias when presented with positive or negative affective words that must be placed into an emotional category. The ability to shift between 2 different spatial aspects (location and direction) is measured in the attentional switching task (AST). The motor screening task (MOT) assesses sensorimotor skill. The Stockings of Cambridge (SOC) task requires spatial planning skills by replicating a visual pattern using the minimum number of moves. The verbal recognition memory (VRM) task assesses the ability to learn, encode, and retrieve new verbal information. The pattern recognition memory (PRM) task measures speed and accuracy of a forced-choice criterion for distinguishing newly or previously presented visual stimuli.

#### WAIS-IV

The WAIS-IV battery assesses more general measurements of cognition and intelligence. The block design subtest measures visual pattern construction abilities. The similarities subtest measures problem solving and conceptualization of how 2 words are subjectively related by the participants. The digit span subtest evaluates accurate recall for a presented sequence of numbers. Nonverbal and abstract problem-solving skills are measured by the matrix reasoning subtest. In the vocabulary subtest, participants must rely on their memory to identify both visually and verbally presented items. The arithmetic subtest assesses the participants’ ability to mentally solve arithmetic problems. The symbol search subtest measures information-processing speed by presenting subjects with target symbols that they must identify when randomized with other symbols. The visual puzzles subtest requires the parti-cipant to use nonverbal reason and verbal perception to reconstruct a visually presented puzzle. Topics of general knowledge are measured in the information subtest. The coding subtest assesses nonverbal learning and nonverbal short-term memory by copying a series of presented symbols.

### Ethics

This study was approved by the institutional review boards at both OU and CU.

### Statistical analyses

Differences in proportions or frequencies as a function of location were tested using a chi-square test. Differences as a function of location for each of the dependent variables in each of the tasks were tested using 2-tailed *t* tests; variables that were expressed as proportions or percentages were transformed prior to analysis using an arcs in-square root transformation to deal with heterogeneity of variance (29). Correlations between measures that either had the same name (e.g., GNG) or that putatively measured the same construct (e.g., working memory) were assessed using the Pearson product moment correlation coefficient, *r*. A final factor analysis was performed on the correlation matrix of the *Z*-transformed values using a varimax rotation. All analyses were performed using SAS 9.4 for Linux (2019; SAS Institute).

## Results

### Demographics

The distribution of subjects by race and ethnicity at each of the 2 locations is presented in **Table 2**. There were no significant differences in race or ethnicity as a function of testing location (χ^2^ = 0.06, NS). Subjects at OU were significantly older (21.2 y; 95% CI: 20.8, 21.8 y) than subjects at CU (20.2 y; 95% CI: 20.0, 20.6 y), although this difference is most likely spurious due to limited variability. There were notable differences as a function of testing location in SES and educational preparation (all data were obtained from the 2014 Fact Books for each university). With respect to SES, whereas 11% of the 2014 entering class at OU had family incomes >$100,000, 50% of the 2014 entering class at CU had family incomes >$100,000. Furthermore, while <1% of the 2014 entering class at OU had family incomes >$250,000, 25% of the 2014 entering class at CU had family incomes >$250,000. With respect to academic achievement, **Figure 1** plots the distribution of SAT (Scholastic Aptitude Test) reading and mathematics scores by testing location and shows that both scores are dominated much more by the high ranges at CU relative to OU (χ^2^ = 271.21, *P* < 0.0001).

**TABLE 2:** Distribution of subjects by race and ethnicity at OU and CU, given as total number (percentage of total)^1^

	OU	CU
Ethnicity		
Non-Hispanic	98 (91)	99 (92)
Hispanic	10 (9)	9 (8)
Total	108	108
Race		
Native American	8 (8)	6 (6)
Asian	25 (23)	38 (35)
African-American	10 (9)	11 (10)
White	65 (60)	53 (49)
Total	108	108

^1^CU, Cornell University; OU, University of Oklahoma.

### Differences as a function of testing location

**Table 3** displays the means, measures of variability, and results of the *t* tests assessing differences due to testing location for all of the dependent measures from each of the tasks in the COGTASKS set of measures. There were no significant differences obtained for any of the dependent measures in this set. **Table 4** presents the same analyses for all of the dependent measures from each of the tasks in the CANTAB set of measures. Significant differences due to testing location were found for the AGNG (accuracy), the MOT, VRM old and new items, and PRM [all reaction time (RT)]. In all cases, performance by CU students was better than that of OU students. **Table 5** presents these analyses for all of the dependent measures from each of the tasks in the WAIS-IV. Significant differences favoring the CU over the OU subjects were obtained for all but 5 of the variables: similarities, digit span, matrix reasoning, and the working memory composite score (with the difference on this measure being marginally significant).

**TABLE 3:** Means, SEMs, 95% CIs, and test statistic (*t*) for tests of differences due to testing location: COGTASKS^1^

	Oklahoma			Cornell			
Task and DV	Mean	SEM	95% CI	Mean	SEM	95% CI	*t*
SRT							
RT (ms)	256	2	251–260	259	2	255–263	−1.15
Accuracy (propn)	0.95	0.001	0.94–0.97	0.97	0.001	0.95–0.97	−1.48
GNG							
RT (ms)	340	4	332–339	348	4	341–355	−1.37
Accuracy (propn)	1.00	0.001	0.99–1.00	0.99	0.008	0.97–1.00	0.48
ANT							
Alerting RT (ms)	31	4	24–38	34	4	27–42	−0.61
Orienting RT (ms)	43	3	37–49	44	4	36–52	−0.16
Conflict RT (ms)	73	4	66–80	74	4	66–82	−0.31
SMS							
Overall accuracy, new items (propn)	0.88	0.01	0.87–0.90	0.86	0.01	0.84–0.88	1.05
Overall accuracy, old items (propn)	0.93	0.01	0.92–0.94	0.92	0.01	0.91–0.93	1.07
RT intercept, new items (ms)	671	22	627–715	722	20	683–761	−1.72
RT intercept, old items (ms)	604	17	571–639	618	17	584–653	−0.56
RT slope, new items (ms)	49	3	43–55	56	3	50–62	−1.75
RT slope, old items (ms)	38	3	32–45	33	3	27–39	1.17
CFE							
d' interaction contrast (SD)	0.29	0.11	0.07–0.54	0.18	0.10	0.02–0.37	0.77
RT interaction contrast (ms)	37	8	21–52	51	8	35–66	1.27
CRT							
d', 4-cue condition (SD)	3.23	0.07	3.09–3.38	3.16	0.08	3.00–3.31	0.73
c, 4-cue condition (SD)	−0.01	0.04	−0.09–0.08	0.04	0.04	−0.04–0.12	−0.88
RT, 4-cue condition, new items (ms)	769	12	745–794	784	11	763–805	−0.91
RT, 4-cue condition, old items (ms)	687	9	670–704	707	8	690–723	−1.64
Percent change in capacity	44.2	3.5	37.3–51.1	36.7	2.5	31.7–41.7	1.75

^1^ANT, attentional network task; c, criterion; CFE, composite face effect; CRT, cued recognition task; DV, dependent variable; d', discriminability; GNG, go/no-go; propn, proportion; RT, reaction time; SD, standard deviation; MS, Sternberg memory search; SRT, simple reaction time.

**TABLE 4:** Means, SEMs, 95% CIs, and test statistic (*t*) for tests of differences due to testing location: CANTAB^1^

	Oklahoma			Cornell			
Task and DV	Mean	SEM	95% CI	Mean	SEM	95% CI	*t*
SRT							
Accuracy (proportion)	1.00			1.00			
RT (ms)	271	8	257–287	263	5	254–272	0.99
AGNG							
Accuracy (proportion)	0.88	0.01	0.86–0.89	0.91	0.01	0.90–0.93	−2.84**
RT (ms)	470	7	456–484	467	5	456–478	0.37
AST							
Acc, non-switch trials (proportion)	0.95	0.003	0.95–0.96	0.96	0.003	0.95–0.96	−0.80
Acc, switch trials (proportion)	0.94	0.005	0.93–0.95	0.95	0.004	0.94–0.96	−1.32
RT, non-switch trials (ms)	502	12	479–526	498	11	476–520	0.29
RT, switch trials (ms)	519	15	490–548	500	14	472–528	0.94
MOT							
Accuracy (proportion)	0.99	0.003	0.98–1.00	0.99	0.004	0.98–0.99	0.91
RT (ms)	966	18	930–1003	815	15	785–845	6.38***
SOC							
Efficiency (min/total moves)	0.90	0.01	0.88–0.92	0.91	0.01	0.59–0.92	−0.74
Deliberation time (min)	12.6	0.6	11.3–13.9	13.1	0.8	11.6–14.6	−0.43
VRM							
d′	3.73	0.05	3.63–3.84	3.78	0.05	3.67–3.89	−0.60
c	0.10	0.03	0.05–0.16	0.07	0.03	0.02–0.13	0.77
Mean RT, old items (ms)	1057	23	1010–1103	953	17	920–986	3.67***
Mean RT, new items (ms)	1029	20	989–1069	958	17	924–992	2.69**
PRM							
Accuracy (proportion)	0.97	0.007	0.96–0.98	0.97	0.007	0.96–0.98	−0.40
Mean RT (ms)	1450	25	1400–1500	1386	24	1339–1433	1.86^+^

^1*^ *P* < 0.05, **P < 0.01, ***P < 0.001, ^+^0.05 < *P* < 0.10. Acc, accuracy; AGNG, affective go/no-go; AST, attentional switching task; MCT, motor control task; PRM, pattern recognition memory; RT, reaction time; SOC, Stockings of Cambridge; SRT, simple reaction time; VRM, verbal recognition memory.

**TABLE 5:** Means, SEMs, 95% CIs, and test statistic (*t*) for tests of differences due to testing location: WAIS-IV^1^

		Oklahoma			Cornell			
Task	DV	Mean	SEM	95% CI	Mean	SEM	95% CI	*t*
Block design	Scaled	11.6	0.31	11.0–12.2	12.9	0.30	12.3–13.4	−2.87**
Similarities	Scaled	11.9	0.29	11.4–12.5	11.4	0.28	10.9–12.0	1.27
Digit span	Scaled	11.8	0.28	11.3–12.4	11.5	0.25	11.0–12.0	0.82
Matrix reasoning	Scaled	11.7	0.24	11.2–12.1	12.0	0.20	11.6–12.4	−1.04
Vocabulary	Scaled	12.5	0.31	11.9–13.1	13.9	0.24	13.4–14.3	−3.53***
Arithmetic	Scaled	11.7	0.29	11.1–12.2	13.0	0.25	12.5–13.5	−3.57***
Symbol search	Scaled	11.6	0.28	11.1–12.2	13.4	0.29	12.8–13.9	−4.32***
Visual puzzles	Scaled	11.1	0.25	10.6–11.6	11.5	0.24	11.1–12.0	−1.37
Information	Scaled	12.3	0.28	11.7–12.8	13.6	0.25	13.1–14.1	−3.62***
Coding	Scaled	12.1	0.28	11.6–12.7	14.4	0.28	13.8–15.0	−5.78***
Verbal comprn	Composite	112.5	1.38	109.7–115.2	116.6	1.15	114.3–118.9	−2.29*
Perc reasoning	Composite	108.3	1.22	105.9–110.7	112.0	1.11	109.8–114.2	−2.24*
Working memory	Composite	109.3	1.36	106.6–112.0	112.4	1.11	110.2–114.6	−1.76^+^
Processing speed	Composite	110.0	1.25	107.5–112.5	121.0	1.29	118.4–123.5	−6.12***
Full score	Scaled	118.0	1.76	114.0–120.9	127.4	1.33	124.8–130.1	−4.56***

^1*^ *P* < 0.05, **P < 0.01, ***P < 0.001, ^+^0.05 < *P* < 0.10. comprn, comprehension; perc, perceptual; WAIS-IV, Weschler Adult Intelligence Scale, fourth edition.

### Correlations between tasks

We next examined the pairwise correlations between tasks that either have the same name or that claim to assess the same cognitive construct. **Table 6** presents the correlation coefficients and shared variances for these pairs of tasks. There were a number of significant correlations, but the majority were weak (all *r *< 0.27), and, on average, the shared variance was only 5% for the pairs of measures having significant correlations. This shared variance is very low relative to the partial variance that some of the COGTASKS variables have demonstrated as a function of treatment condition in some of our field studies (2).

**TABLE 6:** Pairwise Pearson correlations (*r*) and shared variance (*R*^2^) between tasks sharing a common name or putatively measuring the same construct^1^

Task and variable	Correlated with task and variable	*r*	*R* ^2^
COGTASKS SRT	CANTAB SRT	0.26***	0.07
COGTASKS GNG	CANTAB AGNG	0.26***	0.07
COGTASKS ANT Alerting	CANTAB AST RT non-switch	−0.04	0.00
	CANTAB AST RT switch	−0.07	0.01
COGTASKS ANT Orienting	CANTAB AST RT non-switch	0.25***	0.06
	CANTAB AST RT switch	0.15*	0.03
COGTASKS ANT Conflict	CANTAB AST RT non-switch	0.23**	0.05
	CANTAB AST RT switch	0.23**	0.05
COGTASKS SMS accuracy (old items)	WAIS-IV digit span	0.10	0.01
	WAIS-IV working memory index	0.08	0.01
COGTASKS SMS accuracy (new items)	WAIS-IV digit span	0.17*	0.01
	WAIS-IV working memory index	0.16*	0.03
COGTASKS CRT d′	CANTAB VRM d'	−0.07	0.01
	CANTAB PRM accuracy	0.08	0.01
COGTASKS CRT RT (old items)	CANTAB VRM RT (old items)	0.12	0.02
	CANTAB PRM RT	0.15*	0.02
COGTASKS CRT RT (new items)	CANTAB VRM RT (new items)	0.25***	0.07
	CANTAB PRM RT	0.20**	0.04

^1^AGNG, affective go/no-go; ANT, attentional network task; AST, attentional switching task; CANTAB, Cambridge Neuropsychological Test Automated Battery; CRT, cued recognition task; GNG, go/no-go; PRM, pattern recognition memory; RT, reaction time; SMS, Sternberg memory search; SRT, simple reaction time; VRM, visual recognition memory; WAIS-IV, Weschler Adult Intelligence Scale, fourth edition.

### Factor analysis

Finally, we submitted the data to a factor analysis, using the correlation matrix on the *Z*-transformed scores, using a varimax rotation. The first 3 eigenvalues accounted for 40%, 18%, and 14%, respectively, of the variance, cumulatively accounting for 72% of the total variance. The remaining eigenvalues were increasingly <1.0, with the remaining factors each accounting for <2.5% of the variance. The 3-factor solution perfectly segregated the 3 sets of measures, with 1 exception. Factor 1 comprised all of the COGTASK measures, factor 2 comprised all of the WAIS-IV measures (with 1 exception), and factor 3 comprised all of the CANTAB measures. The single exception was the processing speed index from the WAIS-IV, which served to relate all 3 sets of measures.

## Discussion

Accompanying a sustained and increasing interest in assessing cognitive sequelae of a range of nutritional deficiencies and interventions (see Table 1) has been an interest in understanding the best practices in choosing and using behavioral measures of cognition. Although there has been acknowledgment that there is no viable “one size fits all” approach to assessment (7), the field has concentrated on characteristics such as population norms and external validity (12). While these characteristics are desirable, we believe that they have tended to be pursued at the expense of construct validity and biological motivation, often with the thought that if 2 measures share a name or are claimed to be assessing the same cognitive construct, then they must be comparable.

To illustrate the problems with this logic, we conducted what, to our knowledge, is the first and only comparison study in which female participants from 2 US universities completed 3 sets of cognitive assessments: a set of tasks we developed for use in studies of iron repletion (COGTASKS) (1, 4, 14), a widely used commercial neuropsychological battery (CANTAB), and a widely used measure of general intelligence (WAIS-IV). The 2 samples, from 2 US universities, shared (generally) a culture and a language and were very similar in terms of race and ethni-city. However, they were different in terms of SES and academic achievement, 2 factors that are known to affect scores on measures of cognitive performance. All participants completed all 3 sets of assessments.

No significant differences as a function of testing location were found for any of the variables in the COGTASKS, and only a small number of differences were found for the CANTAB variables; however, almost all of the measures from the WAIS-IV had significant differences due to testing location. This suggests that, among these 3 sets of measures, the COGTASKS were the least and WAIS-IV measures were the most sensitive to potentially confounding effects of SES and educational preparation.

Critically, the correlations between tests that either shared the same name or putatively assessed the same construct were uniformly low, with, on average, pairs of measures having only 5% shared variance. Furthermore, a factor analysis on all of the measures simply reproduced their original grouping, with the 3 sets of measures being related by the common factor of processing speed, accounting for 72% of the total variance.

As an example of the weak relations across measures, consider the relation between the GNG task (COGTASKS) and the affective GNG task (CANTAB). Researchers would not be faulted for thinking that these 2 tasks were assessing the same cognitive operations. However, the correlation between the RTs in the 2 tasks was 0.26, with only 7% shared variance. The devil is in the details in this comparison. In the COGTASKS version of the task, simple nonverbal visual forms (vertical and horizontal bars) were used as the go and no-go stimuli. In comparison, in the CANTAB version of the task, the stimuli were words that varied in affective valence. Consequently, even though the 2 tasks had very similar names, and putatively were assessing the same cognitive constructs, the internal computations required by the 2 tasks were quite different. In the COGTASKS version, the test stimulus needed to be properly categorized by way of a learned association, and then a response needed to be either withheld or given. In contrast, in the CANTAB version, retrieval of the word's meaning from semantic memory was required, then the word needed to be properly categorized by a learned association as either a go or a no-go stimulus, all while a potentially interfering or facilitating affective response was being computed. These are 2 very different sets of cognitive and affective operations, so it should not be surprising that the relation between the 2 tasks was rather weak.

We believe that the central conclusion from this work is that, in many cases, construct validity and a concern for underlying biological mechanisms need to be at least as important as population norms and the ability to connect with existing literatures. It all comes down to what needs to be measured. If the questions are at the level of general cognitive functioning, independent of any specific biological state, then packages such as CANTAB and WAIS-IV are very useful. However, if it is the case (as is true with variations in iron status) that the biological state needs to be considered, given the nonuniform distribution of iron in the brain (16), then a more sensitive approach would be to select cognitive assessments based on differential involvement of the specific brain regions that are dependent on iron. The advantages of this more nuanced measurement approach were commented on >30 y ago in the nutrition literature (30) and they remain true. Fortunately, the cognitive neuroscience literature is quite rich, containing multiple sources of evidence helpful in selecting measurements.

Beyond selecting tasks, it is important to understand the nuances of experimental design and data analysis. Returning to the GNG task, simply varying the percentage of go vs. no-go trials can dramatically change the pattern of results. In addition, there are generally accepted practices in analyzing cognitive data that are often left unsaid in publications. For example, when analyzing RTs, it is critical that RTs <200 ms or longer than (for example) 2000 ms be removed from the data, as these reflect anticipatory responses and lapses of attention, respectively. Furthermore, since the distribution of RT data is not Gaussian at the individual subject level, the summary statistic for each subject should be the median rather than the mean and should be calculated either only for correct responses or for correct and error responses separately.

One significant strength of the present study is that the same participants completed all 3 sets of measures, allowing for much stronger inferences regarding the level of shared variance. An additional strength is that the 2 samples differed primarily in terms of SES and educational preparation, 2 factors that are known to influence scores on cognitive tests. This allowed us to quantify the extent to which each set of tasks would be subject to the potentially confounding influences of differences in SES and educational preparation. One weakness of the present study is that, relative to the general population, the 2 samples of college students can be assumed to be higher performing, which does pose some limits to generalizability. That being noted, the level of performance on the COGTASKS was comparable to what we have obtained with college-aged women in Rwanda (2), male and female adolescents in India (4), and women of reproductive age in India (14). A second potential weakness is that the inferences drawn here are limited to tests of the cognitive constructs represented in the overlap among the 3 sets of tasks. A third weakness is that the results, drawn as they are from a healthy population, do not necessarily generalize to individuals with specific dietary insufficiencies. However, we would suggest that the present findings suggest similar results with other cognitive constructs (e.g., measures of executive function).

Perhaps, then, the last conclusion to be drawn is that this is an area that can benefit immensely from interdisciplinary collaborations. Certainly, it has been our experience that cross-talk between nutritional science and cognitive neuroscience has been quite fruitful. We would further argue that it has allowed for measurement that is more biologically grounded and stronger in terms of construct validity than would have been the case otherwise.

## Notes

This project was funded by a grant from HarvestPlus International.

Author disclosures: The authors report no conflicts of interest.

Abbreviations used: AGNG, affective go/no-go; ANT, attentional network task; AST, attentional switching task; CANTAB, Cambridge Neuropsychological Test Automated Battery; COGTASKS, cognitive task battery; CU, Cornell University; CRT, cued recognition task; GNG, go/no-go; MOT, motor screening task; OU, University of Oklahoma; PRM, pattern recognition memory; RT, reaction time; SES, socioeconomic status; SRT, simple reaction time; VRM, visual recognition memory; WAIS-IV, Weschler Adult Intelligence Scale, fourth edition.

## References

[1] Murray-Kolb LE , Wenger MJ, Scott SP, Rhoten SE, Lung'aho MG, Haas JD. Consumption of iron-biofortified beans positively affects cognitive performance in 18-to 27-year-old Rwandan female college students in an 18-week randomized controlled efficacy trial. J Nutr. 2017;147(11):2109–17. 28954841 10.3945/jn.117.255356 PMC5657139

[2] Wenger MJ , Rhoten SE, Murray-Kolb LE, Scott SP, Boy E, Gahutu J-B, Haas JD. Changes in iron status are related to changes in brain activity and behavior in Rwandan female university students: Results from a randomized controlled efficacy trial involving iron-biofortified beans. J Nutr. 2019;149(4):687–97. 30926992 10.1093/jn/nxy265 PMC6461719

[3] Salami A , Avelar-Pereira B, Garzón B, Sitnikov R, Kalpouzos G. Functional coherence of striatal resting-state networks is modulated by striatal iron content. Neuroimage. 2018;183:495–503. 30125714 10.1016/j.neuroimage.2018.08.036

[4] Scott SP , Murray-Kolb LE, Wenger MJ, Udipi SA, Ghugre PS, Boy E, Haas JD. Cognitive performance in Indian school-going adolescents is positively affected by consumption of iron-biofortified pearl millet: a 6-month randomized controlled efficacy trial. J Nutr. 2018;148(9):1462–71. 30016516 10.1093/jn/nxy113

[5] Caudill MA , Strupp BJ, Muscalu L, Nevins JE, Canfield RL. Maternal choline supplementation during the third trimester of pregnancy improves infant information processing speed: a randomized, double-blind, controlled feeding study. FASEB J. 2018;32(4):2172–80. 29217669 10.1096/fj.201700692RR PMC6988845

[6] Hajiluian G , Abbasalizad Farhangi M, Nameni G, Shahabi P, Megari-Abbasi M. Oxidative stress-induced cognitive impairment in obesity can be reversed by vitamin D administration in rats. Nutr Neurosci. 2018;21(10):744–52. 28683595 10.1080/1028415X.2017.1348436

[7] Edson E , Dunfee, R, Heyman M. Landscape analysis of brain development tests. McClean, VA: Booz Allen Hamilton; 2012.

[8] Neath I . Human memory: an introduction to research, data, and theory. Independence, KY: Thomson Brooks/Cole Publishing Co; 1998.

[9] Kandel ER , Schwartz JH, Jessell TM. Principles of neural science. New York: McGraw-Hill; 2000.

[10] Forster KI , Forster JC. DMDX: a Windows display program with millisecond accuracy. Behav Res Methods Instrum Comput. 2003;35(1):116–24. 12723786 10.3758/bf03195503

[11] Falkingham M , Abdelhamid A, Curtis P, Fairweather-Tait S, Dye L, Hooper L. The effects of oral iron supplementation on cognition in older children and adults: a systematic review and meta-analysis. Nutr J. 2010;9(1):4. 20100340 10.1186/1475-2891-9-4 PMC2831810

[12] Cook RL , O'Dwyer NJ, Parker HM, Donges CE, Cheng HL, Steinbeck KS, Cox EP, Franklin JL, Garg ML, Rooney KB. Iron deficiency anemia, not iron deficiency, is associated with reduced attention in healthy young women. Nutrients. 2017;9(11):1216. 10.3390/nu9111216 PMC5707688 29113086

[13] Greig AJ , Patterson AJ, Collins CE, Chalmers KA. Iron deficiency, cognition, mental health and fatigue in women of childbearing age: a systematic review. J Nutr Sci. 2013;2:e14. 25191562 10.1017/jns.2013.7 PMC4153327

[14] Wenger MJ , Murray-Kolb LE, Nevins JE, Venkatramanan S, Reinhart GA, Wesley A, Haas JD. Consumption of a double-fortified salt affects perceptual, attentional, and mnemonic functioning in women in a randomized controlled trial in India. J Nutr. 2017;147(12):2297–308. 29021371 10.3945/jn.117.251587 PMC6519426

[15] Beard JL , Connor JR. Iron status and neural functioning. Annu Rev Nutr. 2003;23(1):41–58. 12704220 10.1146/annurev.nutr.23.020102.075739

[16] Connor J , Menzies S, Martin SS, Mufson E. Cellular distribution of transferrin, ferritin, and iron in normal and aged human brains. J Neurosci Res. 1990;27(4):595–611. 2079720 10.1002/jnr.490270421

[17] Lee R , Singh L, van Liefde D, Callaghan-Gillespie M, Steiner-Asiedu M, Saalia K, Edwards C, Serena A, Hershey T, Manary MJ. Milk powder added to a school meal increases cognitive test scores in Ghanaian children. J Nutr. 2018;148(7):1177–84. 29905824 10.1093/jn/nxy083

[18] Lelijveld N , Jalloh AA, Kampondeni SD, Seal A, Wells JC, Goyheneix M, Chimwezi E, Mallewa M, Nyirenda MJ, Heyderman RS. Brain MRI and cognitive function seven years after surviving an episode of severe acute malnutrition in a cohort of Malawian children. Public Health Nutr. 2019;22(8):1406–14. 30501662 10.1017/S1368980018003282 PMC6411134

[19] Robinson SM , Crozier SR, Miles EA, Gale CR, Calder PC, Cooper C, Inskip HM, Godfrey KM. Preconception maternal iodine status is positively associated with IQ but not with measures of executive function in childhood. J Nutr. 2018;148(6):959–66. 29767745 10.1093/jn/nxy054 PMC5991217

[20] Mohorko N , Černelič-Bizjak M, Poklar-Vatovec T, Grom G, Kenig S, Petelin A, Jenko-Pražnikar Z. Weight loss, improved physical performance, cognitive function, eating behavior, and metabolic profile in a 12-week ketogenic diet in obese adults. Nutr Res. 2019;62:64–77. 30803508 10.1016/j.nutres.2018.11.007

[21] Bermejo-Pareja F , Llamas-Velasco S, Tapias-Merino E, Hernández Gallego J, Hernández-Cabria M, Collado-Yurrita L, López-Arrieta JM. Is milk and dairy intake a preventive factor for elderly cognition (dementia and Alzheimer's)? A quality review of cohort surveys. Nutr Rev. 2020; [epub ahead of print] 14 December, 2020, doi.org/10.1093/nutrit/nuaa045. 10.1093/nutrit/nuaa045 33316068

[22] Ma F , Li Q, Zhou X, Zhao J, Song A, Li W, Liu H, Xu W, Huang G. Effects of folic acid supplementation on cognitive function and Aβ-related biomarkers in mild cognitive impairment: a randomized controlled trial. Eur J Nutr. 2019;58(1):345–56. 29255930 10.1007/s00394-017-1598-5

[23] Sirin SR . Socioeconomic status and academic achievement: a meta-analytic review of research. Rev Educ Res. 2005;75(3):417–53.

[24] Ebbinghaus H . Über das gedächtnis: untersuchungen zur experimentellen psychologie. Berlin: Duncker & Humblot; 1885.

[25] Fan J , McCandliss BD, Sommer T, Raz A, Posner MI. Testing the efficiency and independence of attentional networks. J Cogn Neurosci. 2002;14(3):340–7. 11970796 10.1162/089892902317361886

[26] Sternberg S . High-speed scanning in human memory. Science. 1966;153(3736):652–4. 5939936 10.1126/science.153.3736.652

[27] Murphy J , Gray KL, Cook R. The composite face illusion. Psychon Bull Rev. 2017;24(2):245–61. 27488558 10.3758/s13423-016-1131-5

[28] Wenger MJ , Negash S, Petersen RC, Petersen L. Modeling and estimating recall processing capacity: sensitivity and diagnostic utility in application to mild cognitive impairment. J Math Psych. 2010;54(1):73–89. 10.1016/j.jmp.2009.04.012 PMC2861301 20436932

[29] Zar J . Biostatistical analysis. 5th ed. Upper Saddle River, NJ: Pearson Education; 2010.

[30] Haas JD , Fairchild MW. Summary and conclusions of the International Conference on Iron Deficiency and Behavioral Development, October 10–12, 1988. Am J Clin Nutr. 1989;50(3):703–5. 2773837

